# The risk of a second cancer after hospitalisation for venous thromboembolism

**DOI:** 10.1038/sj.bjc.6602757

**Published:** 2005-08-30

**Authors:** H T Sørensen, L Pedersen, L Mellemkjær, S P Johnsen, M V Skriver, J H Olsen, J A Baron

**Affiliations:** 1 The Department of Clinical Epidemiology, Aarhus University Hospital, Ole Worms Allé 150, 8000 Aarhus C, Denmark; 2 The Danish Cancer Society, Institute of Cancer Epidemiology, Strandboulevarden 49, 2100 Copenhagen Ø, Denmark; 3 Departments of Medicine and Community and Family Medicine, Dartmouth Medical School, Hanover, NH 03755-3861, USA

**Keywords:** venous thromboembolism, epidemiology, prognosis, risk

## Abstract

Although venous thromboembolism (VTE) is common in patients with cancer, it is not known if it is associated with risk of a second malignancy. Using the Danish Cancer Registry and National Registry of Patients, we studied a population-based cohort of 6285 patients with cancer who had an episode of VTE. The risk of a second cancer was compared with that among 30 713 cancer patients without VTE, matched for age, sex, cancer site and year of diagnosis. Overall, the relative risk for a second cancer diagnosis was 1.3 (95% confidence interval (CI) 1.1–1.4). However, the excess risk varied with the time from the initial cancer diagnosis to the thrombotic event. If the thrombotic episode occurred within the first year, the relative risk for a second cancer was 1.0 (95% CI 0.9–1.3), but if the VTE occurred more than 1 year after the initial cancer, the overall relative risk for a second cancer was 1.4 (95% CI 1.2–1.7), with strong associations for cancers of the digestive organs, ovary and prostate. The association between VTE and subsequent incident cancer extends to patients who already have had a cancer diagnosis.

The association between cancer and venous thrombosis has been recognised for more than 100 years (Piccioli *et al*, 1996), since episodic migratory thrombophlebitis was first reported in patients with cancer by Trousseau (Sørensen and Baron, 2005). Patients with clinically overt cancer may develop venous thromboembolism (VTE) at any stage of the disease (Agnelli, 1997; Rickles and Levine, 2001), aggravated by surgery, chemotherapy and intravenous catheters (Rickles and Levine, 2001). Occasionally, the thromboembolic event may occur before the clinical presentation of the cancer, and it is well known that the risk of a first cancer diagnosis is greatly increased in the year immediately after VTE (Prandoni *et al*, 1992; Baron *et al*, 1998; Sørensen *et al*, 1998, 2000; Murchison *et al*, 2004).

The implications of cancer risk subsequent to venous thrombosis for patients with a previous cancer are less clear. An episode of VTE is a marker of a poor cancer prognosis (Sørensen *et al*, 2000), but it is not known if this is associated with an increased risk of a second malignancy, as in patients without prevalent cancer. Since this may have important clinical implications for the care of cancer patients, we investigated the risk of a second cancer in patients with a known malignancy who experienced an episode of deep venous thrombosis or pulmonary embolism, using population-based data from the Danish Cancer Registry and National Registry of Patients.

## MATERIALS AND METHODS

This population-based study was based on a cohort of 45 201 patients with VTE identified through the Danish National Registry of Patients. This registry, established in 1977, includes information on 99.4% of all admissions to Danish acute care nonpsychiatric hospitals (Andersen *et al*, 1999). Recorded information includes the civil registration number (unique to each Danish citizen), the dates of admission and discharge, the surgical procedures performed and up to 20 discharge diagnoses, classified according to the Danish version of the International Classification of Diseases, 8th edition (ICD-8) (Andersen *et al*, 1999) until the end of 1993, and ICD-10 thereafter. It is possible to obtain the full discharge history of a patient by linking discharge records with the civil registration number. Study subjects were identified by searching for patients who had a first time discharge diagnosis of either lower limb deep venous thrombosis or pulmonary embolism (ICD-8 codes 451.00 and 450.99; ICD-10 codes I26, I80.1, I80.2 and I80.3) during at least one hospitalisation between 1 January 1977 and 31 December 1999.

To identify members of the VTE cohort with prevalent cancers (other than nonmelanoma skin cancer), we used civil registration numbers to link them to the Danish Cancer Registry (Storm *et al*, 1997). Here, cancers are classified according to the modified Danish version of the ICD, 7th Revision. Registration is based on notification forms completed by hospital departments and practicing physicians whenever a case of cancer is diagnosed or found at autopsy and whenever there are changes in an initial diagnosis. The Registry has been in operation since 1943 and is 95–98% complete and valid (Storm *et al*, 1997). In the VTE cancer cohort, we identified 10 107 patients who had had a cancer diagnosis prior to the VTE hospitalisation (4711 with deep venous thrombosis, 5312 with pulmonary embolism and 84 with both diagnoses): 3822 patients died during the hospital admission for VTE, leaving 6285 patients for follow-up.

As a control cohort, we selected from the Cancer Registry patients with a primary cancer diagnosis but without evidence of VTE in the National Registry of Patients. For each VTE cancer case, five cancer controls were identified, matched on age (within 5 years), sex, primary cancer site and year of cancer diagnosis. We required each control to be alive on the discharge date of the corresponding case for VTE.

### Statistical analysis

Members of both study cohorts were linked through their civil registration numbers to the nationwide Danish Civil Registration System (with electronic records on vital status, including dates of emigration and death for the entire Danish population), and to the Danish Cancer Registry to identify subsequent deaths and cancer diagnoses. Both cohorts were followed from the date of the case VTE hospitalisation until the date of a second cancer (other than nonmelanoma skin cancer), censoring from death or emigration, or end of follow-up (31 December 1999), whichever came first. We estimated the relative risk by comparing the incidence of a second primary cancer between the VTE cancer patients and the cancer controls, using rate ratios with 95% confidence intervals (CI) computed from proportional-hazard regression models. We also computed these rate ratios within two strata of time between the first primary cancer and the thrombosis hospitalisation (a year or less, *vs* more than 1 year). We estimated cumulative risks using life-table methods.

## RESULTS

The mean age of the 6285 members of the VTE cancer cohort was 68.6 years (standard deviation 12.2); 46.1% were men. The cancer sites most heavily represented in the cohort were breast (13.8%), colon (10.0%), prostate (9.6%), lymphatic system (9.1%), urinary tract (7.7%), rectum (6.8%), lung (6.8%) and corpus uteri (5.6%). In 974 patients (15.5%), the first episode of VTE occurred within a month of the initial cancer diagnosis.

A total of 343 second cancers were diagnosed in the VTE cancer cohort compared with 1981 in the control cancer cohort, yielding a relative risk during the entire follow-up of 1.3 (95% CI 1.1–1.4). The relative risks were slightly lower for those over 70 years of age, 1.2 (95% CI 1.1–1.4) than for those younger, 1.4 (95% CI 1.2–1.7), but were almost identical for men and women (data not shown). For the various second malignancies, relative risks for cancers of the upper gastrointestinal tract, ovary and prostate were particularly increased (Table 1).

**Figure 1:**
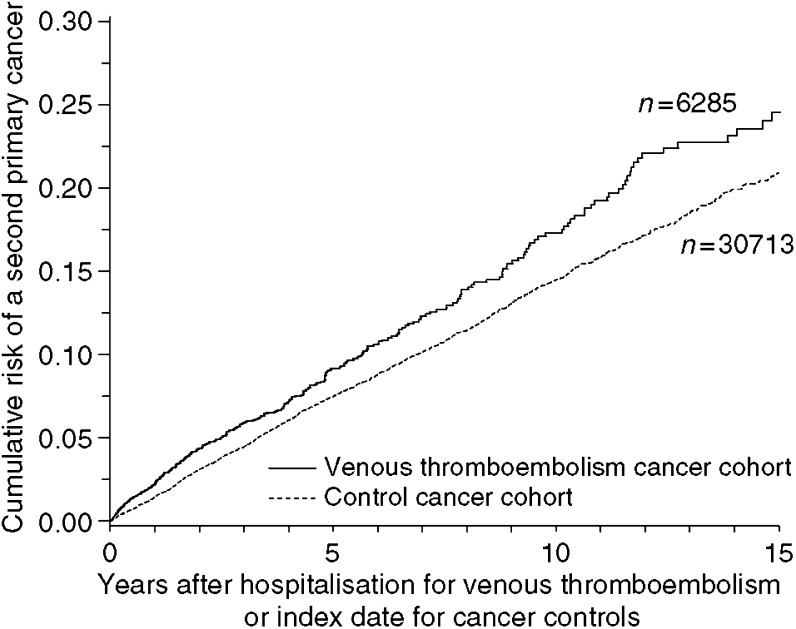
Cumulative risk of a second cancer among cancer cases with venous thromboembolism and cancer controls.

Figure 1 shows the evolution over time of the cumulative risk of a second cancer in the two cohorts. In the first year after the VTE, there was a second cancer diagnosis for 2.3% of the VTE cancer patients and for 1.4% of cancer controls (relative risk 1.6, 95% CI 1.3–2.0) (Table 1).

Of the 3339 VTE cancer cohort members alive 1 year after the thrombotic event and without a second cancer up to that time, 238 had a diagnosis of a malignancy at a later time (years 2–23 of follow-up) *vs* 1610 second cancers among 21 713 1-year survivors in the control cancer cohort (relative risk 1.1, 95% CI 1.0–1.3) (Table 2). There was little variation over time in the relative risks during this period (data not shown). Cancer of the prostate and colorectal cancer were the only cancer sites with significantly elevated thrombosis-associated risks that persisted beyond the first year of follow-up.

The risk of a second cancer varied with the interval between the first cancer and the VTE episode. Patients with a thromboembolic event more than a year after their cancer diagnosis had a 40% increase in risk for a second cancer (relative risk 1.4, 95% CI 1.2–1.7). The relative risk of a second cancer was slightly higher (relative risk 1.7, 95% CI 1.3–2.2) in the first year after the thrombotic event, but there was also a clear increase in cancer risk even over more prolonged follow-up (relative risk 1.4, 95% CI 1.2–1.6) (Table 3). In contrast, cancer patients hospitalised with VTE within 1 year of the first cancer diagnosis had exactly the same overall risk of a subsequent second cancer as controls (relative risk 1.0, 95% CI 0.9–1.3). These patients with early thrombosis did have an increased cancer risk in the first year after the VTE (relative risk 1.6; 95% CI 1.1–2.3), but this was followed by a subsequent period of slightly lower cancer risk, which brought the overall relative risk to unity (Table 3).

Table 4 shows the cumulative 1-year risks of a second primary cancer in subgroups of patients with VTE more than 1 year after the first cancer. Particularly, high risks of a second cancer were seen for patients with kidney and bladder cancer (1 year cumulative risk 4.5%, 95% CI 2.3–6.7).

## DISCUSSION

In this large nationwide follow-up study of cancer patients, we found an increased risk of second cancers associated with VTE, largely among patients whose thrombotic episode occurred 1 or more years after the first primary cancer. Risks of cancers of the ovary, prostate, hepatobiliary tract and pancreas were increased in the first year after the thrombosis diagnosis; there was a longer-term substantial increase in risk only for prostate and colorectal cancer.

The associations we observed with second cancers parallels that for a first malignancy, in which risk is also increased soon after a thrombotic episode (Baron *et al*, 1998; Sørensen *et al*, 1998; Prandoni, 2002). The spectrum of thrombosis-associated second cancers is very similar to that for first cancers (Baron *et al*, 1998; Sørensen *et al*, 1998), and thus similar aetiologic factors may be at play.

Among patients with early VTE – within a year of the first cancer diagnosis – increased surveillance is likely to be a factor in explaining the pattern of increased risks followed by decreased risks. The absence of such a pattern for patients with a later thrombotic episode suggests that diagnostic bias is probably not a factor, and implies that the association in this group has a biological basis. The variation over time in the relative risks provides some clues regarding this issue.

It is implausible that VTE or its treatment could cause a second solid tumour to develop within a year or two. Indeed, if the thrombotic event somehow contributed to the aetiology of the second cancer, we would have expected the relative risks to increase with follow-up, reflecting the long latency period of most epithelial cancers (Baron *et al*, 1998). If, on the other hand, shared risk factors were underlying the association, a more or less constant excess risk over time would be expected, as seen among patients with a thrombotic episode more than 1 year after the first cancer diagnosis. These risk factors could theoretically be smoking, obesity and hormone replacement therapy, since these factors are suggested or established risk factors for both VTE and cancer (Baron *et al*, 1998). However, the cancers with the increased relative risks in association with venous thrombosis – ovary, prostate, liver, biliary and pancreas – do not prominently share these lifestyle risk factors. On balance, it is most likely that the second cancer was occult and caused the venous thrombosis, conceivably through changes in the clotting pathway (Silverstein and Nachman, 1992; Zacharski *et al*, 1992).

Our study has both strengths and limitations. The large population we studied was well defined and the long-term follow-up complete, because our design relied on computerised registries with complete nationwide coverage. This prevented selection bias and gave us a relatively high statistical precision. It is also well known that many cancers are associated with an increased risk of a second malignancy (Curtis *et al*, 1994; Leone *et al*, 1999; Levi *et al*, 1999; Brenner *et al*, 2000), and we therefore used other patients with incident cancers as controls. Our use of routine data underlies another strength: since the study itself did not affect the diagnostic process, it could not introduce surveillance bias in follow-up. On the other hand, we identified cases of VTE through an administrative database, the Danish National Registry of Patients, which may not be entirely accurate. This misclassification has been estimated to approximately 8–20% in Sweden and the US (Kniffin *et al*, 1994; Schulman and Lindmarker, 2000). Any misclassification of VTE in the hospital discharge records would cause bias towards the null hypothesis. As noted above, differential surveillance might also play a role.

The absolute risk of a second cancer after thromboembolism is relatively low (about 2% over the first year), and so the benefit of screening for a second cancer in cancer/thrombosis patients seems limited. Detection of a second malignancy would require an extensive work-up with high costs to detect a relatively small number of cancers. Moreover, the sites of the second malignancies are not those for which effective screening programmes have been devised and it is unclear if screening for a second cancer in this setting would change prognosis.

Over the past 20 years, there has been increasing clinical evidence that anticoagulants have antitumour effects reducing the risk of cancer in patients with VTE and improving survival in patients with advanced malignancy (Zacharski *et al*, 1981; Schulman and Lindmarker, 2000; Kakkar *et al*, 2004). Cancer patients with VTE are at increased risk of second malignancies, but further work is needed before specific guidelines for their management would be appropriate.

## Figures and Tables

**Table 1:** Relative risk (rate ratio) of a second cancer among thrombosis cancer patients and control cancer patients in the first year of follow-up

	**VTE cancer cohort, *N*=6285**		**Control cancer cohort, *N*=30 713**		
**Sites of second cancer (ICD-7 code)^a^**	**No. of events**	**No./1000 person-year**	**No. of events**	**No./1000 person-year**	**Relative risk (95% CI)**
All	105	24.3	371	14.5	1.6 (1.3–2.0)
Oesophagus (150)	3	0.7	8	0.3	2.2 (0.6–8.4)
Colon, rectum (153, 154)	13	3.0	55	2.2	1.4 (0.8–2.5)
Liver, primary, gall bladder, pancreas (155.0, 155.1, 157)	13	3.0	23	0.9	3.2 (1.6–6.4)
Lung, primary (162)	16	3.7	67	2.6	1.4 (0.8–2.4)
Breast (170)	7	2.2	181	1.8	1.3 (0.9–1.9)
Ovary (175)	5	1.2	6	0.2	5.0 (1.5–16.3)
Prostate (177)	11	2.5	29	1.1	2.2 (1.1–4.3)
Kidney, bladder (180, 181)	13	3.0	48	1.9	1.6 (0.9–2.9)
Lymphatic and haematological (200–205)	5	1.2	20	0.8	1.5 (0.6–4.0)

VTE=venous thromboembolism; CI=confidence interval.

a Modified version of the 7th International Classification of Diseases.

**Table 2:** Relative risk (rate ratio) of a second cancer of selected sites among thrombosis cancer patients and control cancer patients during 2–23 years of follow-up

	**VTE cancer cohort, *N*=3339**		**Control cancer cohort, *N*=21 713**		
**Sites of second cancer (ICD-7 code)^a^**	**No. of events**	**No./1000 person-year**	**No. of events**	**No./1000 person-year**	**Relative risk (95% CI)**
All	238	18.2	1610	15.8	1.1 (1.0–1.3)
Oesophagus (150)	4	0.3	22	0.2	1.4 (0.5–4.0)
Colon, rectum (153, 154)	47	3.6	254	2.5	1.4 (1.1–2.0)
Liver, primary, gall bladder, pancreas (155.0, 155.1, 157)	11	0.8	89	0.9	0.9 (0.5–1.8)
Lung, primary (162)	30	2.3	276	2.7	0.8 (0.6–1.2)
Breast (170)	29	2.2	181	1.8	1.3 (0.9–1.9)
Ovary (175)	3	0.2	33	0.3	0.7 (0.2–2.3)
Prostate (177)	30	2.3	121	1.2	1.8 (1.2–2.7)
Kidney, bladder (180, 181)	22	1.7	194	1.9	0.9 (0.6–1.3)
Lymphatic and haematological (200–205)	18	1.4	129	1.3	1.1 (0.7–1.8)

VTE=venous thromboembolism; CI=confidence interval.

a Modified version of the 7th International Classification of Diseases.

**Table 3:** Relative risk (rate ratio) of a second cancer diagnosis (*n*=343) among cancer patients (*n*=6285) with venous thromboembolism by time after first cancer diagnosis

		**Follow-up interval**		
**Interval between first cancer and VTE**	**Number of VTE cancer patients/controls**	**First year**	**1+ years**	**Overall**
Overall	6285/30 713	1.6 (1.3–2.0)	1.1 (1.0–1.3)	1.3 (1.1–1.4)
0–1 year	3081/14 896	1.6 (1.1–2.3)	0.9 (0.7–1.1)	1.0 (0.9–1.3)
>1 year	3204/15 817	1.7 (1.3–2.2)	1.4 (1.2–1.6)	1.4 (1.2–1.7)

VTE=venous thromboembolism.

**Table 4:** Cumulative 1-year risk of a second cancer in patients with an episode of VTE more than 1 year after the first cancer

**Variable**	**Number of subjects at risk**	**Cumulative absolute risk^a^**
All	3204	2.7 (2.1–3.3)
Women	1764	2.6 (1.7–3.4)
Men	1440	2.9 (1.9–3.8)
< 70 years	1386	2.9 (1.9–3.9)
⩾70 years	1818	2.7 (1.8–3.5)

*Site of first cancer (ICD-7 code)*		
Colon, rectum (153, 154)	489	2.9 (1.9–3.9)
Breast (170)	607	1.2 (0.2–2.2)
Prostate (177)	352	2.7 (0.7–4.7)
Kidney, bladder (180, 181)	423	4.5 (2.3–6.7)
Lymphatic and haematological (200–205)	261	2.4 (0.3–4.5)

Other	1072	3.3 (2.0–4.5)

VTE=venous thromboembolism; ICD-7=7th International Classification of Diseases.

a Percentage.
